# Supplementary figures and images for: Dynamics of the Gut Microbiome in Shigella-Infected Children during the First Two Years of Life
Source: mSystems. 2022 Sep 19;7(5):e00442-22. doi: 10.1128/msystems.00442-22 (PMC9600951; doi:10.1128/msystems.00442-22)

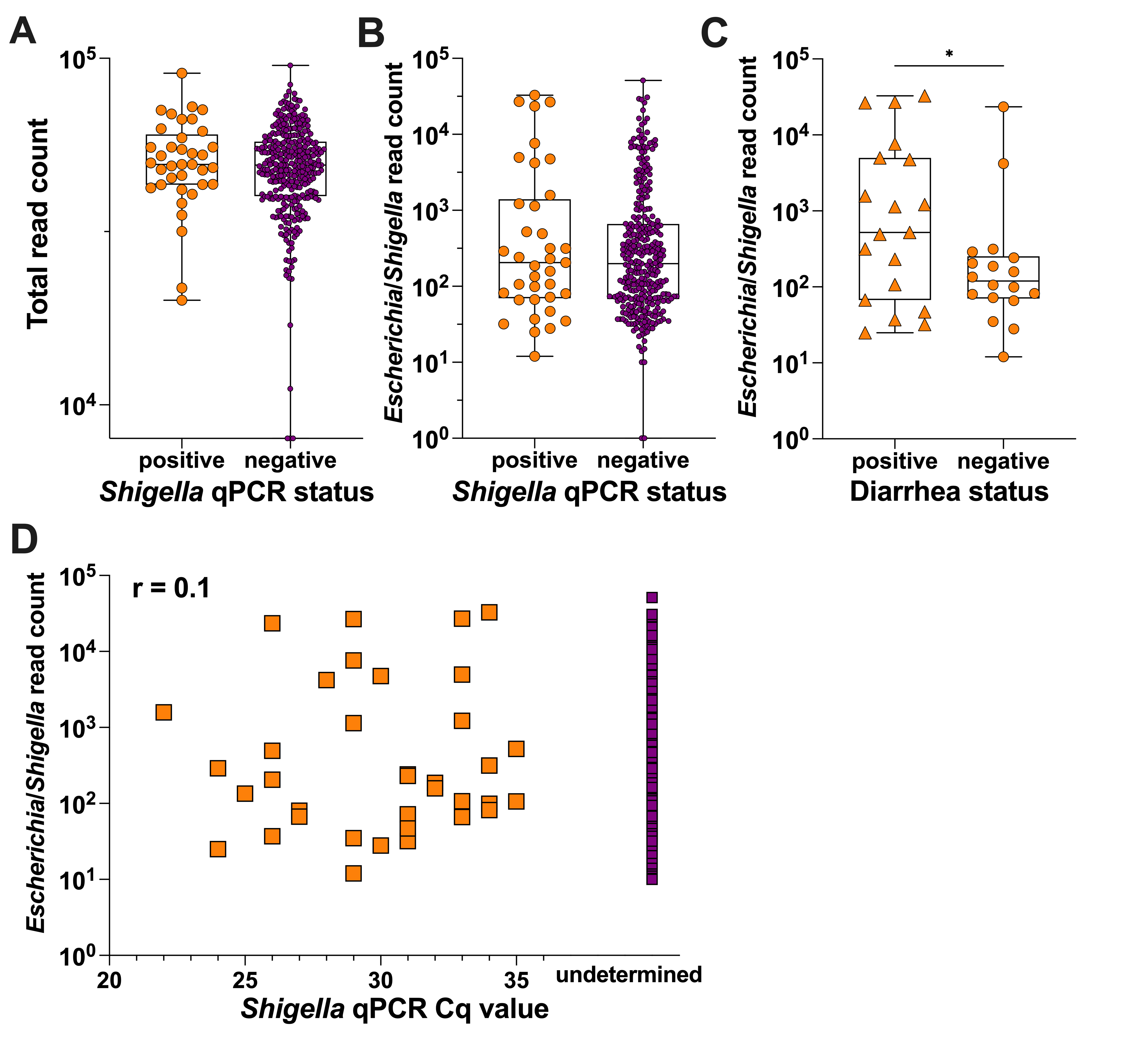

Supplement: FIG S1 [file msystems.00442-22-s0006.tif]

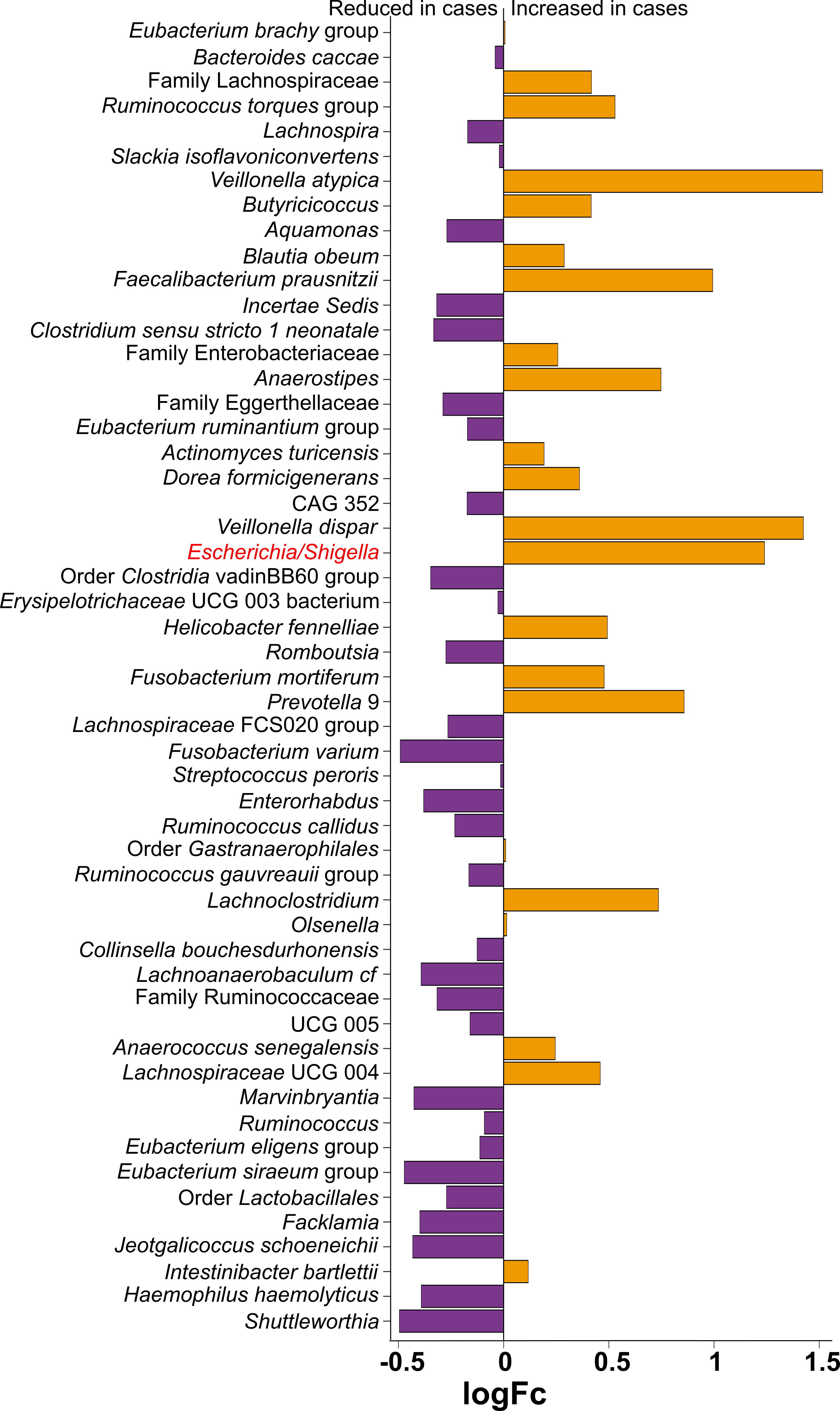

Supplement: FIG S2 [file msystems.00442-22-s0007.tif]

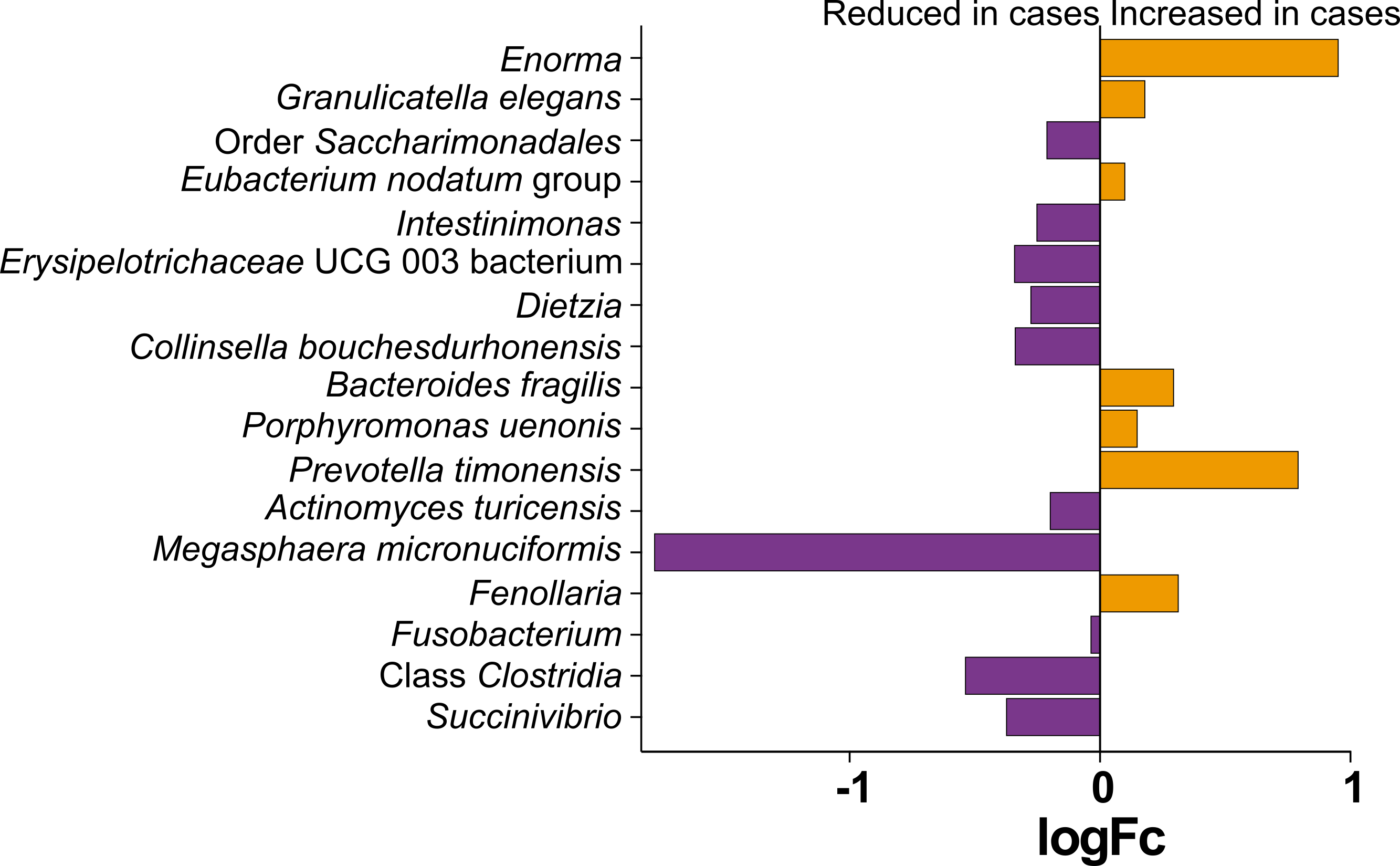

Supplement: FIG S3 [file msystems.00442-22-s0008.tif]

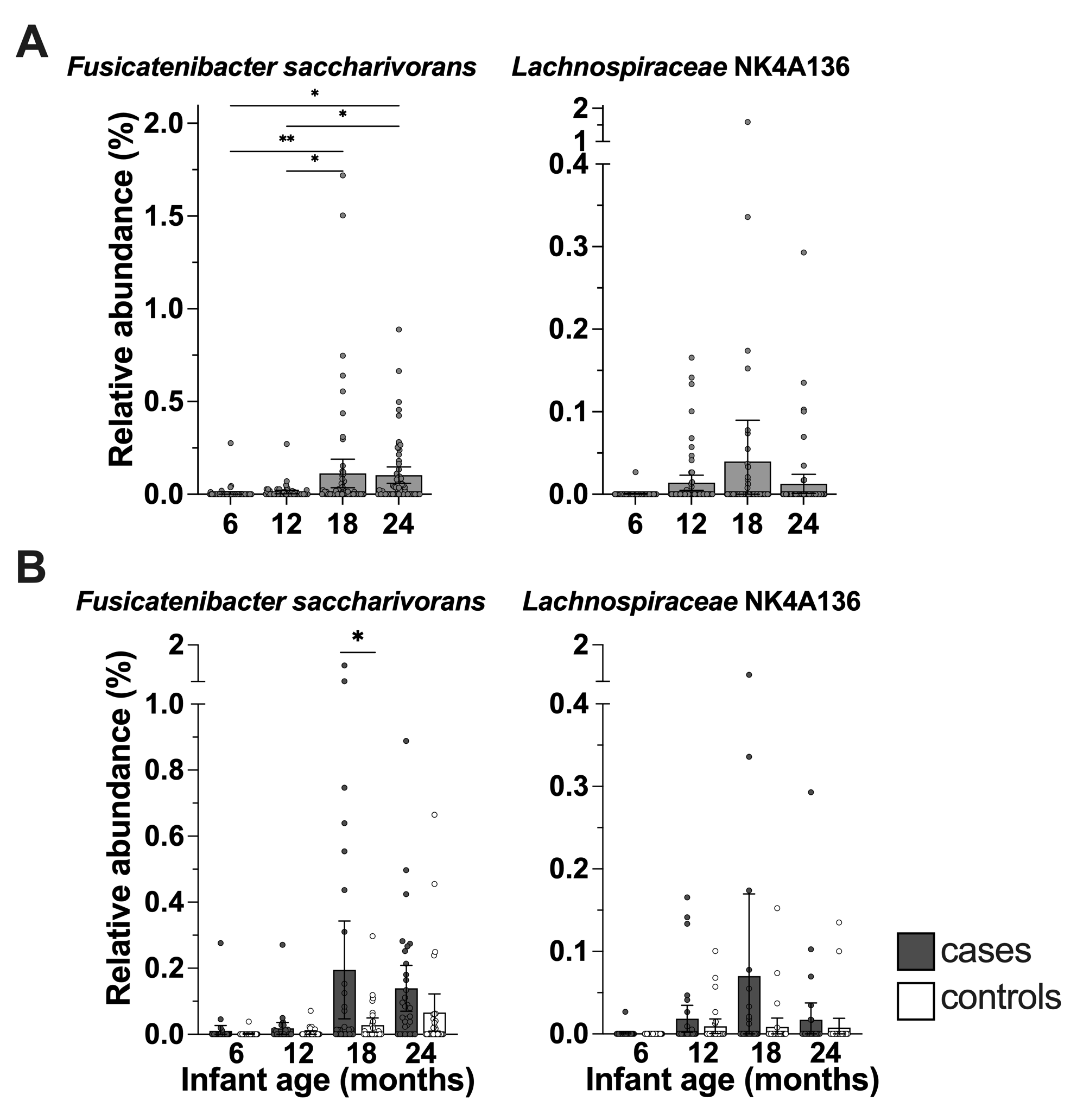

Supplement: FIG S5 [file msystems.00442-22-s0010.tif]

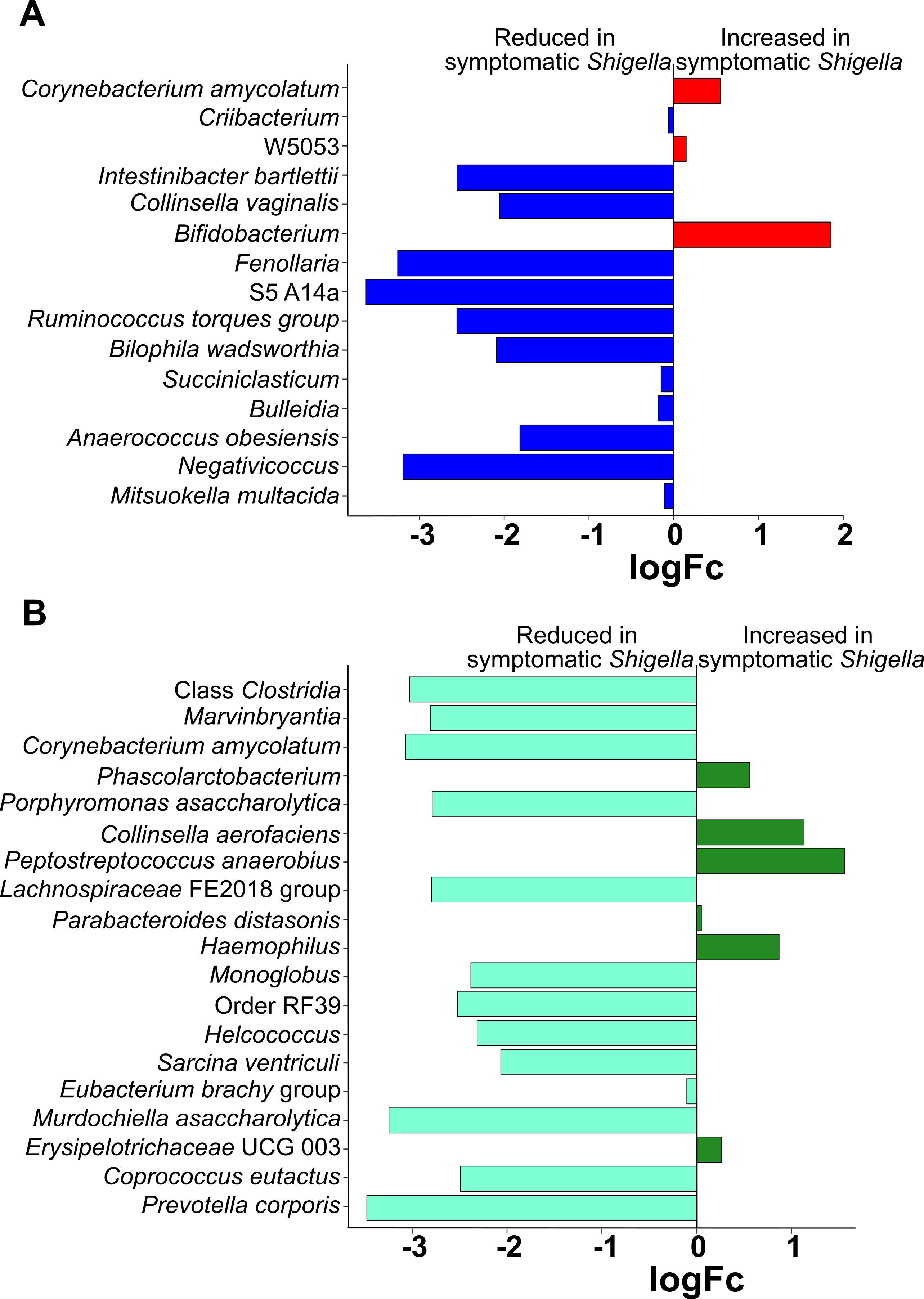

Supplement: FIG S4 [file msystems.00442-22-s0009.tif]
