# Supplementary material for: Dynamics of the Gut Microbiome in Shigella-Infected Children during the First Two Years of Life
Source: mSystems. 2022 Sep 19;7(5):e00442-22. doi: 10.1128/msystems.00442-22 (PMC9600951; doi:10.1128/msystems.00442-22)
Supplement: TABLE S1 [file msystems.00442-22-s0002.docx]

| **Table S1. Mean relative abundance of top 10 taxa** | | | | |
| --- | --- | --- | --- | --- |
| Taxa | Month 6  Mean (%) (95%CI) | Month 12  Mean (%) (95%CI) | Month 18  Mean (%) (95%CI) | Month 24  Mean (%) (95%CI) |
| *Bifidobacterium longum* | 13.3 (10.0-16.7) | 3.7 (2.3-5.2) | 1.9 (1.1-2.7) | 0.7(0.2-1.1) |
| *Prevotella* | 10.8 (8.8-12.8) | 7.6 (5.7-9.5) | 7.7 (6.1-9.2) | 6.3 (4.8-7.9) |
| *Prevotella* 9 | 2.4 (1.0-3.8) | 7.4 (5.0-9.7) | 6.1 (4.6-7.6) | 9.7 (7.2-12.2) |
| *Finegoldia magna* | 5.6 (4.1-7.1) | 6.3 (4.7-8.0) | 5.4 (3.8-7.0) | 3.7 (2.3-5.0) |
| *Peptoniphilus* | 5.6 (4.5-6.6) | 5.8 (4.8-6.8) | 6.2 (5.2-7.1) | 5.1 (4.2-6.0) |
| *Anaerococcus* | 3.6 (2.6-4.5) | 3.6 (2.9-4.4) | 5.4 (4.0-6.8) | 5.6 (4.1-7.0) |
| *Escherichia/*  *Shigella* | 4.4 (2.4-6.5) | 2.0 (0-4.3) | 0.9 (0.3-1.5) | 0.6 (0.1-1.0) |
| *Bifidobacterium kashiwanohense* | 0.8 (0.4-1.0) | 4.8 (3.4-6.1) | 3.8 (2.7-4.9) | 3.5 (2.3-4.7) |
| *Anaerococcus vaginalis* | 4.0 (2.4-5.5) | 5.1 (3.6-6.6) | 3.4 (2.6-4.2) | 2.4 (1.8-2.9) |
| *Streptococcus* | 2.6 (0.8-4.5) | 2.8 (1.1-4.4) | 2.4 (1.2-3.6) | 0.7 (0.3-1.1) |
