# Supplementary material for: Dynamics of the Gut Microbiome in Shigella-Infected Children during the First Two Years of Life
Source: mSystems. 2022 Sep 19;7(5):e00442-22. doi: 10.1128/msystems.00442-22 (PMC9600951; doi:10.1128/msystems.00442-22)
Supplement: TABLE S2 [file msystems.00442-22-s0003.docx]

| **Table S2. Cross sectional comparison between case index visits and matched control index visits** | | |
| --- | --- | --- |
|  | F value^2^ | Pr(>F)^3^ |
| Case status^1^ | 0.43 | 0.52 |
| Diarrhea positivity | 6.85 | **0.012** |
| Infant age | 21.26 | **<0.001** |
| Infant sex | 0.98 | 0.33 |
| Month of year | 1.00 | 0.46 |
| Short-term antibiotics administration^4^ | 6.17 | **0.017** |
| ^1^Cases- infants with at least one *Shigella* qPCR positive sample; ^2^Controls- infants with no *Shigella* qPCR positive samples  ^2^Variation between sample mean relative to variation within samples  ^3^Pr(>F) values test comparisons between cases and controls using ANOVA (Type III test)  ^4^Short-term refers to antibiotics given within 14 days before sample collection | | |
