# Supplementary material for: Dynamics of the Gut Microbiome in Shigella-Infected Children during the First Two Years of Life
Source: mSystems. 2022 Sep 19;7(5):e00442-22. doi: 10.1128/msystems.00442-22 (PMC9600951; doi:10.1128/msystems.00442-22)
Supplement: TABLE S3 [file msystems.00442-22-s0004.docx]

| **Table S3. Comparisons of SDI between cases and controls** | | | | |
| --- | --- | --- | --- | --- |
|  | Mean SDI  cases^1^ | Mean SDI controls^2^ | F/z value | P value^3^ |
| *Shigella* infection | | |  |  |
| before index case | 2.82 | 2.76 | 1.01 | 0.32 |
| at index case | 2.90 | 3.16 | 0.43 | 0.52 |
| after index case | 3.29 | 3.18 | 0.03 | 0.98 |
| Symptomatic vs asymptomatic *Shigella* infection | | | | |
| before infection | 2.68 | 2.96 | -1.15 | 0.25 |
| after infection | 3.24 | 3.41 | -0.46 | 0.65 |
| *Shigella*-driven vs other-cause diarrhea | | | | |
| at index case | 2.58 | 2.82 | 0.68 | 0.42 |
| after infection | 3.07 | 3.04 | 0.07 | 0.95 |
| ^1^Cases- infants with at least one *Shigella* qPCR positive sample  ^2^Controls- infants with no *Shigella* qPCR positive samples  ^3^P values represent either Pr(>F) values testing comparisons between cases and controls using ANOVA (Type III test), or Pr(>z) values testing comparisons between cases and controls by fitting a generalized linear or logistic mixed-effects models | | | | |
